# Capturing the diversity of working age life-courses: A European perspective on cohorts born before 1945

**DOI:** 10.1371/journal.pone.0212400

**Published:** 2019-02-22

**Authors:** Kathrin Komp-Leukkunen

**Affiliations:** Faculty of Social Sciences, University of Helsinki, Helsinki, Finland; Universitat Luzern, SWITZERLAND

## Abstract

Life-courses describe people’s activities from the cradle to the grave. Because life-courses are typically complex, models are used to simplify their description. The most commonly used model is tripartite, representing lives in subsequent periods of education, work, and retirement. However, researchers criticize this model as limited in the activities considered, overly simplistic in the activity sequence, and blind to variation between life-courses. This article explores working age life-courses, which typically show high diversity. Multichannel sequence and cluster analyses are conducted on people’s activities from age 15 to 65. Data stem from the life-history interviews of the Survey of Health, Ageing and Retirement in Europe, capturing cohorts born before 1945. Findings show that three out of four working age life-courses are in line with the tripartite model. This share is particularly high among men, the cohort born 1935 to 1944, and in Northern and Eastern Europe. In contrast, a considerable share of women spent their working age on homemaking, especially women born before 1935, and those living in Southern Europe. Finally, a smaller number of men spent their working age on paid work, followed by a period of illness or of non-employment. The working age life-course patterns identified are used to develop alternative life-course models. However, for a parsimonious solution, the use of two models suffices. A combination of the tripartite model and the model equating middle age to homemaking captures the lives of more than nine out of ten older Europeans. The prevalence of working age life-course patterns in a population is country-specific, and the country differences align with the welfare regimes. This perspective makes working age life-courses characteristics of a society that can be used to map social inequalities at the macro-level and capture social change over time.

## Introduction

Life-courses are a central concept in the social sciences. They describe how human lives develop from the cradle to the grave, highlighting activities that people engage in and the experiences that are important to them [1]. These life-courses are used to describe and explain developments over time, showing that human lives unfold according to characteristic patterns and dynamics [2–4]. Additionally, life-courses are used to pinpoint where people are in their personal development process, drawing on characteristics that are typical for different phases of the life-course [1, 5, 6]. Because of their broad applicability, researchers interested in all age groups and in a variety of topics use life-courses to study, for example, the effects of childhood living conditions, the participation in further education at different ages, and pathways to financial well-being in retirement [7–11].

Life-courses are typically complex because individuals simultaneously engage in multiple activities, lives change over time, and people interact with each other and with the institutions around them [12]. Therefore, researchers need to introduce some simplification to describe life-courses in their entirety. A means of choice is the use of a model, which is a simplified portrayal of reality, highlighting only some main features [13]. Martin Kohli [12, 14–16] devised today’s most widely used life-course model. He argues that modern Western societies developed institutions which give life-courses a distinct structure, thereby turning the life-course itself into an institution. Because modern Western societies are centred on paid work, the life-course is also structured around paid work. It has a tripartite structure: youth is dedicated to socialization and education, which prepares for paid work; middle age is when people work for pay; old age is the time of retirement, after people have withdrawn from paid work. This life-course model allows researchers and practitioners to understand life-course structures at a glance, and it enables researchers to more easily compare life-courses within and across countries and over time [17–20]. Moreover, it is the basis for the calculation of dependency ratios, which are widely used indicators for the progression of population aging and the reform pressure on pay-as-you-go financed pension schemes [21, 22]. These ratios are numerical representations of the relation between dependent and independent individuals in a society, equating middle-agers to independent individuals, and youths and older people to dependent individuals [23, 24].

Although the tripartite life-course model is widely used, it is also widely disputed. Researchers stress the diversity of life-courses [20, 25–28], thereby casting doubt on the accuracy of any single life-course model. This diversity is particularly pronounced during working age, when women strive to balance work and having a family, and when men may experience spells of unemployment [27, 29]. Consequently, alternative life-course models would especially need to reflect the diverse experiences during working age. Unfortunately, the criticism arose from studies that were either conceptual in nature [30], focused on a short period of time only [25, 31], or investigated only one aspect of the life-course, such as the degree of de-standardization [28] or the intersection of work with partnership and parenthood [27]. While these studies clearly pinpoint the need for alternative life-course models, they do not identify these models.

The present article fills this lacunae. It explores the structure of working age life-courses, tracing Europeans from age 15 to 65 with life-history data from the Survey of Health, Ageing and Retirement in Europe. In doing so, it answers four research questions. First, which working age life-courses exist? Second, how do working age life-courses differ across gender? Third, how do they differ across cohorts? (Previous studies identified gender and cohorts are the prominent reasons for within-country life-course differences.) And fourth, how do working age life-courses differ across countries? This last question draws on the idea that life-courses differ with social context, such as labor market structures and culture. This study uses the concept of welfare regimes to structure the country differences observed. Welfare regimes describe ideal types of welfare states, which also create regime-specific life-course patterns. The findings are used to determine which alternative life-course models are needed to better capture diversity during working age. The within- and across-country differences identified pinpoint in which social groups the alternative life-course models primarily occur.

### Within-country differences in the life-course model

**Fig 1:**
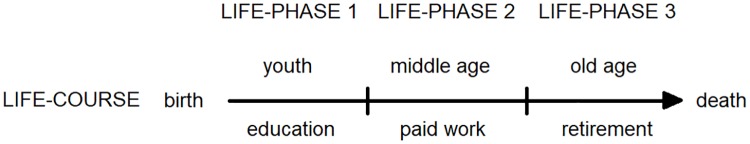
The tripartite life-course model.

The simplicity of the tripartite life-course model is both its strength and its Achilles’ heel. Kohli [12, 14–16] devised this model mainly through conceptual considerations, assigning activities to youth, middle age, and old age. This step turns youth, middle age, and old age into life-phases, which are longer-lasting situations in a person’s life that are dedicated to characteristic activities [1, 26] (see Fig 1). The tripartite model can capture life-courses best if they are institutionalized and standardized. The institutionalization of life-courses means that regulations such as compulsory schooling and set retirement ages are in place, regulating people’s activities [12]. Standardization means that people’s life-courses all follow the same pattern, instead of following unique logics [12]. If life-courses are only weakly institutionalized or de-standardized then the tripartite model may be inaccurate, and alternative life-courses emerge.

Reports about the diversity of working age life-courses highlight an incomplete institutionalization and standardization of middle age. The middle age is defined as the time when people work for pay, but it is institutionalized as the time when youth protection does not prevent them from working and when pension regulations do not yet allow them to retire from work. Therefore, middle age is a time when people could work for pay, but it is not necessarily a time when they do so. A prominent reason why middle-agers do not work is that they cannot find employment, therefore experiencing spells of unemployment or non-employment [32]. Another prominent reason is that they have insufficient time for paid work, for example because they are running a household or raising young children [33]. Middle-agers have to develop their own strategies for meeting these challenges, which leads to a de-standardization of the life-course. Diversity during working age emerges. Consequently, the first hypothesis is: Working age life-courses are structured around paid work, around a fluctuation in and out of the labor force, or around efforts to balance work and family life.

Previous research clearly established social differences in life-courses, most notably across gender and birth cohorts. Gender differences arise because culturally engrained gender roles guide decisions and activities [34–36]. Women are the main care providers to kin and they shoulder the majority of household chores, which requires them to balance paid work with homemaking during working age [37–40]. As a result, their working age life-courses can be structured around paid work, homemaking, or a combination of both. In contrast, men’s working age life-courses are usually structured around paid work, with homemaking playing a negligible role [29, 41, 42]. Therefore, the second hypothesis is: Female working age life-courses are structured around paid work or a combination of work and homemaking, whereas male working age life-courses are structured around paid work or a fluctuation in and out of the workforce. The third hypothesis is: Working age life-courses that are structured around paid work are more common among men than among women.

The gender differences evolved with the birth cohorts, meaning according to when people were born. Cohorts experience historical events and social structures at the same time, which gives their lives similar life-course structures and differentiates them from other cohorts [20, 26, 43, 44]. Previous studies suggest that female labor force participation rates and the institutionalization of the life-course increase hand-in-hand [12, 45]. The logic is simple: the more women work for pay, the more life-courses are shaped by labor market institutions. Of course women still need to coordinate their workforce participation with homemaking and caregiving, which keeps their working age life-courses volatile and multidimensional [9, 46]. As a result, diversity in working age life-courses persists. However, the general trend is clear: the younger cohort sees fewer women in working age life-courses that are structured around homemaking (Hypothesis 4). Moreover, the younger cohort sees new patterns of working age life-courses (Hypotheses 5), because of women’s increasing efforts to combine paid work and homemaking.

Among men, the trend is reversed. In some countries unemployment rates increased and many governments sought to delay retirement [47–50]. Increasing unemployment rates can lead individuals to fluctuate in and out of the labor market, giving workforce participation a more episodic character. Moreover, they can cause youths to go through a spell of unemployment or non-employment before entering the labor market, and they can cause older workers to experience similar spells before retiring [19, 29, 32, 51, 52]. In their efforts to delay retirement, many governments increased the pensionable age and closed pathways to early retirement [48, 50]. As a result, some older individuals who would otherwise have retired early now remain unemployed or non-employed until they reach the pensionable age [53–55]. Therefore, the younger cohort has more men fluctuating in and out of the labor market during working age (Hypothesis 6).

### Between-country differences in the life-course model

Life-courses differ across countries in marked ways because they reflect country-specific cultures, histories, and institutions [56, 57]. Their strong link to country characteristics turns them into properties of social systems [12, 58]. Mayer [20, 26] suggested that the country differences in life-courses align with welfare regimes. Welfare regimes are ideal types of welfare states, which are characterized through politics, welfare policies, and their resulting social structures [59]. Several studies showed that these welfare regimes also align with country differences in gender roles [60–63]. Table 1 shows what the welfare regimes imply for working age life-courses [12], [20], [26], [59], [60], [64], [65], [66].

**Table 1:** Welfare regimes and working age life-courses.

	Welfare regime				
	Social-democratic	Liberal	Conservative	Rudimentary	Post-paternalistic
**Country examples**	DK, S	CH, IE	A, B, F, D, NL	GR, I, E	CZ, PL
**Welfare state**	universalist, strong state	strong market & inequalities	family & state cooperate	family centered	ex-communist, developing
**Gender difference**	low	intermediate	intermediate	high	intermediate
**Cohort difference**	low	intermediate	intermediate	intermediate	high
**Diversity in life-courses**	lowest	intermediate, higher: women	intermediate, higher: women	highest	intermediate, higher: young cohort

The country abbreviations are A ‘Austria’, B ‘Belgium’, CZ ‘Czech Republic’, DK ‘Denmark’, F ‘France’, D ‘Germany’, GR ‘Greece’, I ‘Italy’, IE ‘Ireland’, NL ‘Netherlands’, PL ‘Poland’, E ‘Spain’, S ‘Sweden’, and CH ‘Switzerland’

The social-democratic regime is common among Northern European countries, of which Denmark and Sweden are included in this study. This regime is characterized by a strong welfare state that follows universalist ideas and facilitates equality among its citizens [59]. Its well-developed social services allow women to combine work and childcare more easily than the other regimes do, thereby leading to high female workforce participation rates [60, 62]. Overall, this regime is characterized by a high labor market integration of its citizens [59, 61, 62]. This suggests it has the most homogenous working age life-courses and the highest share of working age life-courses structured around paid work (Hypothesis 7).

The liberal regime is typical for most Anglo-Saxon countries, and Switzerland, with the latter and Ireland being part of this study. This type of regime favors market mechanisms for welfare production and accepts the resulting social inequalities [59]. The strong reliance on the market drives the citizens of this regime to participate in paid work [26, 59]. However, those with high incomes or wealth can access a wide range of services, which can allow them to retire early and their partner to withdraw from paid work. As a result, gender differences in workforce participation emerge [60, 62, 67]. Therefore, the eighth hypothesis is: The liberal regime has an intermediate share of individuals whose working age life-course is structured around paid work.

The conservative regime is prevalent in central Europe, for example in Austria, Belgium, France, and Germany [26, 59]. Because of its approach to gender roles, the Netherlands also belongs to this regime [60]. The conservative regime splits the responsibility for its citizens’ welfare between the state and families, leaving women to shoulder a considerable number of care tasks [59, 60, 63]. As a result, women participate in the labor force only to a limited extent, often either in part-time employment or in interrupted careers [26, 63]. Many countries of this regime previously countered structural unemployment by encouraging early retirement, also via disability and long-term unemployment benefits, but have limited these options during recent years [47, 50]. This regime has an intermediate share of working age life-courses that are structured around paid work, and this share is considerably lower among women (Hypothesis 9).

The rudimentary regime is typical for Southern European countries such as Greece, Italy, and Spain. It emphasizes the role of families even more than the conservative regime does, while providing fewer public services [60, 64]. As a consequence, women in this regime adhere to more traditional gender roles and spend a large amount of time on family care and homemaking [33, 61]. Therefore, the tenth hypothesis is: The share of working age life-courses structured around paid work is lowest in the rudimentary regime, and the share of women’s working age life-courses that are structured around homemaking is highest.

Finally, the post-paternalistic regime is found in Eastern European countries such as the Czech Republic and Poland. These countries had planned economies under communist rule, which provided continuous working careers for all citizens [65, 66]. When the communist period ended, unemployment emerged as a new phenomenon. Additionally, individuals gained the possibility to abstain from paid work if they wished to do so, for example, to look after their home and children [66, 68]. Therefore, there is an intermediate share of working age life-courses that are structured around paid work in the post-paternalistic regime, and this share is be considerably higher in the older cohort (Hypothesis 11).

## Material and methods

### Data

Data stem from wave 3 of the Survey of Health, Ageing and Retirement in Europe (SHARE). SHARE is a panel study that contains information on the employment, health status, social situation, and activities of Europeans aged 50+ years, with the data being collected every other year since 2004 [69]. Wave 3 of SHARE (collected in 2008/09) additionally contains life-history data, which describe the development of the respondents’ entire working careers, family, and health histories. The data collection has been reviewed and approved by the Ethics Committee of the University of Mannheim, and all participants provided written consent [70]. This wave used event history calendars to ensure an accurate recollection [71]. Wave 3 was collected in 14 countries: Austria, Belgium, the Czech Republic, Denmark, France, Germany, Greece, Italy, Ireland, the Netherlands, Poland, Spain, Sweden, and Switzerland. I selected those respondents from wave 3 that were aged 65 years or older. There were 15 229 respondents of this age in the dataset. Then, I excluded respondents with missing values on any variable. The dataset contained 270 respondents with missing values, which is less than 2 percent. An analysis of the missing values suggests that data was missing at random [72]. I excluded these respondents because of their low number and because the exclusion created no bias in the dataset. The final dataset contains 14 959 cases. Table 2 displays the final number of cases by country and gender.

**Table 2:** Number, gender, and birth cohort of respondents, by country.

	Men			Women		
	Number of cases	Born until 1934 (%)	Born 1935–44 (%)	Number of cases	Born until 1934 (%)	Born 1935–44 (%)
**Austria**	256	34.4	65.6	371	42.9	57.1
**Belgium**	680	48.4	51.5	853	50.9	49.1
**Czech Republic**	403	37.5	62.5	515	39.4	60.6
**Denmark**	473	40.4	59.6	550	49.8	50.2
**France**	545	47.9	52.1	748	50.4	49.6
**Germany**	533	31.9	68.1	538	36.8	63.2
**Greece**	644	42.2	57.8	791	48.0	52.0
**Italy**	724	39.5	60.5	733	38.2	61.8
**Ireland**	184	39.1	60.9	225	42.2	57.8
**Netherlands**	537	37.6	62.4	571	38.9	61.1
**Poland**	397	41.3	58.7	449	45.4	54.6
**Spain**	600	53.3	46.7	753	52.3	47.7
**Sweden**	549	40.8	59.2	629	41.3	58.7
**Switzerland**	321	44.2	55.8	387	46.8	53.2
**Total**	6 846	42.0	58.0	8 113	45.1	54.9

### Variables

The analyses use variables describing people’s socio-demographic characteristics and their activities. Variables capturing people’s socio-demographic characteristics are gender (‘male’/’female’), birth cohort (‘until 1934’; aged 75+ during data collection/‘1935–1944’; aged 65–74 during data collection) and country of residence (‘Austria’/‘Belgium’/‘Czech Republic’/‘Denmark’/‘France’/‘Germany’/‘Greece’/‘Italy’/‘Ireland’/‘Netherlands’/‘Poland’/‘Spain’/‘Sweden’/‘Switzerland’). Overall, the sample includes slightly more women than men and slightly more women from the younger cohort (see Table 2), which reflects the population structure in Europe [73].

People’s activities are captured in four variables, which are measured annually. The annual measurements indicate what happened each year from age 15 to age 65, thereby showing the working age life-course. The first variable captures the labor market status, with the answer categories being: ‘working’, ‘unemployed’, ‘retired and not working’, ‘retired and working’ and ‘non-employed’. The second variable shows whether the respondent is a homemaker (‘yes’/‘no’). The third variable describes whether the respondent is in education (‘yes’/‘no’). The fourth variable explores whether the respondent is in good health (‘yes’/‘no’). For this variable, the respondents described their self-assessed health status, considering sickness as well as disability. Table 3 shows the number of years that men and women spend on each of these activities. The men in this sample spent most of their time working (almost 80 percent), followed by some time being non-employed (about 20 percent). Education, illness, and homemaking played little role for them. In contrast, the women in this sample spent about half of their time in non-employment, followed by working (about 40 percent). Moreover, they were homemaking for one third of the time. Also among women, illness and education took up only a small amount of time.

**Table 3:** Frequencies of activities by gender, pooled time-series (in percent).

		Men	Women
**Labor market status**	Working	78.7	44.0
	Unemployed	1.2	2.0
	Retired, not working	0.5	0.5
	Retired & working	0.3	0.1
	Non-employed	19.3	53.4
**Homemaking**	Is homemaking	0.7	34.9
	Is not homemaking	99.3	65.1
**Educational status**	In education or training	5.7	3.8
	Not in education or training	94.3	96.2
**Health status**	In good health	98.4	98.6
	Not in good health	1.6	1.4

### Analytic strategy

The data analysis starts out with sequence analyses, which are followed by cluster analyses. A sequence analysis maps activities over time, showing in which order they occurred and how long they lasted [74, 75]. Thereby, it portrays the working age life-course, as individuals had described it in their life-history interviews. This article conducts multichannel sequence analyses, which analyze several activities that occur at the same time. The activities analyzed are the labor market status, being a homemaker, educational participation, and health status. The choice of multichannel sequence analyses accounts for the fact that people can engage in several activities at the same time, which is one aspect that the tripartite life-course model neglects [76]. In this article, the sequence analyses map the activities from age 15 to age 65, meaning during working age, where people experience most changes and fluctuations in activities [77, 78]. The sequence analyses uses dynamic optimal matching distance, which has the advantage of using data-based–and therefore objective–substitution costs [29]. Then, cluster analyses using Ward’s method compare the life-courses and measure their similarities, suggesting which clusters they can best be grouped in. Sequence analyses are often carried out together with cluster analyses, because sequence analyses produce complex information that needs to be condensed before it can be interpreted–and cluster analyses can perform such a condensation of information [25, 74]. The analyses are carried out for men and women separately, because there are marked gender differences in activities in some countries [27, 37, 41]. All analyses were carried out with the statistics software R, using the packages TraMineR, cluster, foreign and graphicsQC. In a last step, the clusters are described more closely through cross-tables with the respondents’ gender, cohort, and country of residence.

## Results

**Fig 2:**
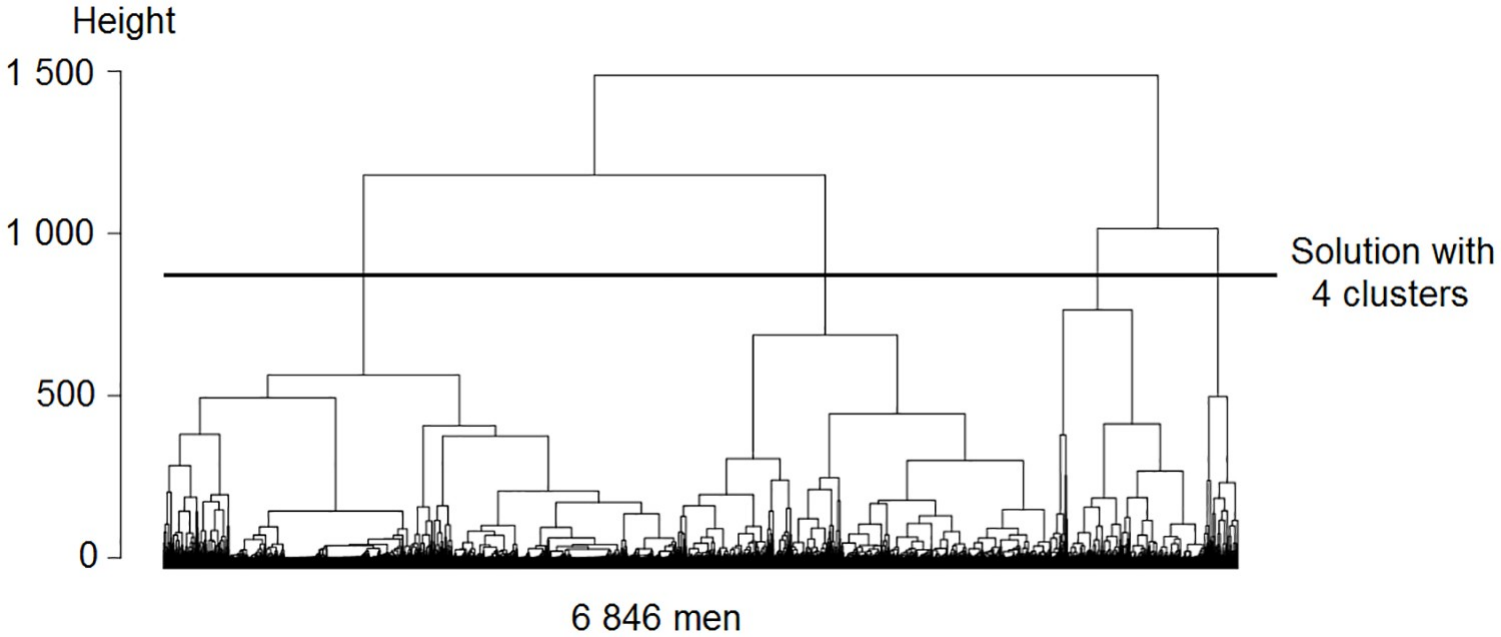
Dendrogram for the clusters among men. The model fit is 0.36 Point Biserial Correlation, 0.26 Average Silhouette Width, 1 872 Calinski-Harabasz index using squared distances, 0.51 Hubert’s Somers’ D, and 0.19 Hubert’s C.

**Fig 3:**
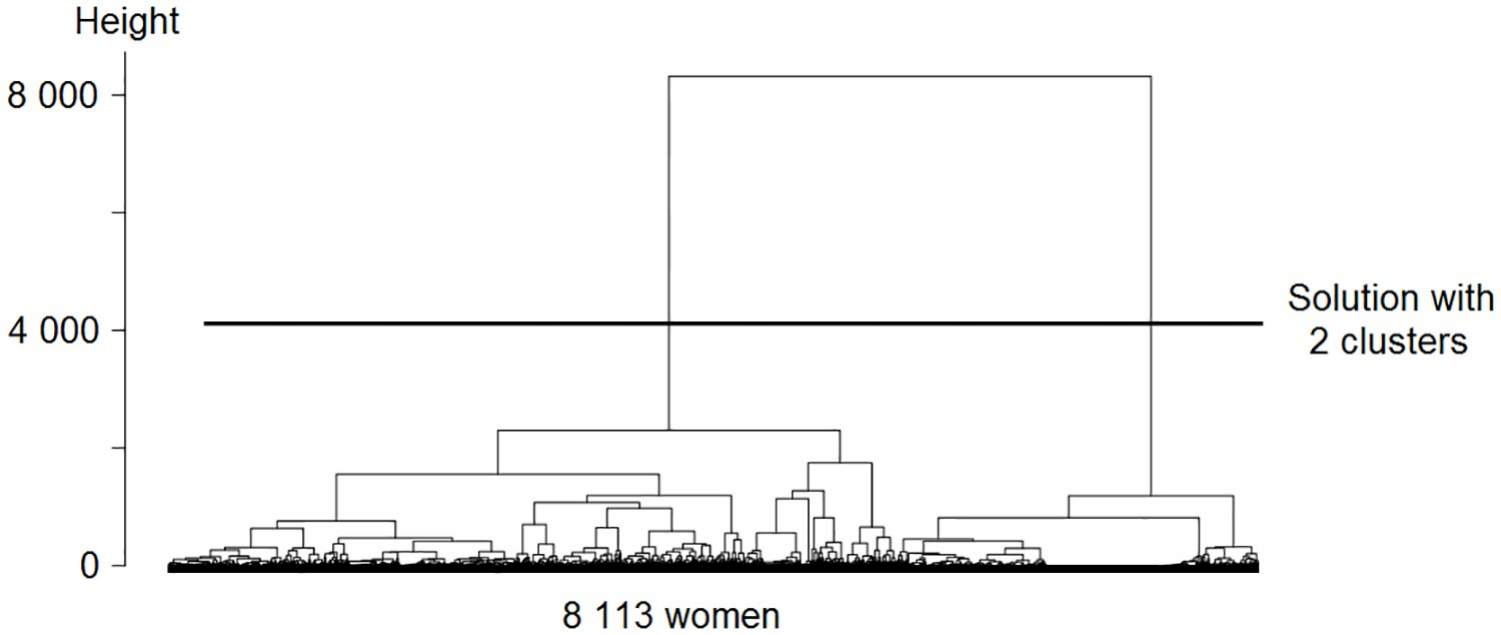
Dendrogram for the clusters among women. The model fit is 0.79 Point Biserial Correlation, 0.61 Average Silhouette Width, 16 932 Calinski-Harabasz index using squared distances, 0.91 Hubert’s Somers’ D, and 0.05 Hubert’s C.

The cluster analyses show how many types of working age life-course patterns the data holds. The dendrograms in Figs 2 and 3 display this information. A dendrogram is a tree diagram, showing into how many clusters the sample can be split, and how similar the clusters are to one another. The top of the dendrogram represents a situation where the entire sample is one cluster, and the bottom represents a situation where each case is a cluster [79]. The dendrogram shows the distance between clusters in terms of height: the further two clusters are apart from one another, the higher their connecting node is above them. The vertical axis on the left-hand side of the dendrogram provides a scale for the height [80]. The dendrograms show that women follow two clearly distinct working age life-course patterns, whereas men follow four working age life-course patterns that differ less than those of women. The clearer distinction among women is reflected in the greater height of the women’s dendrogram, which amounts to 8 000 units as compared to 1 500 units among men. Moreover, it is reflected in the better model fit for the clustering among women on all model fit parameters: the Point Biserial Correlation, Average Silhouette Width, and Hubert’s Somers’ D are closer to 1, the Calinski-Harabasz index using squared distances is higher, and Hubert’s C is lower [81].

**Fig 4:**
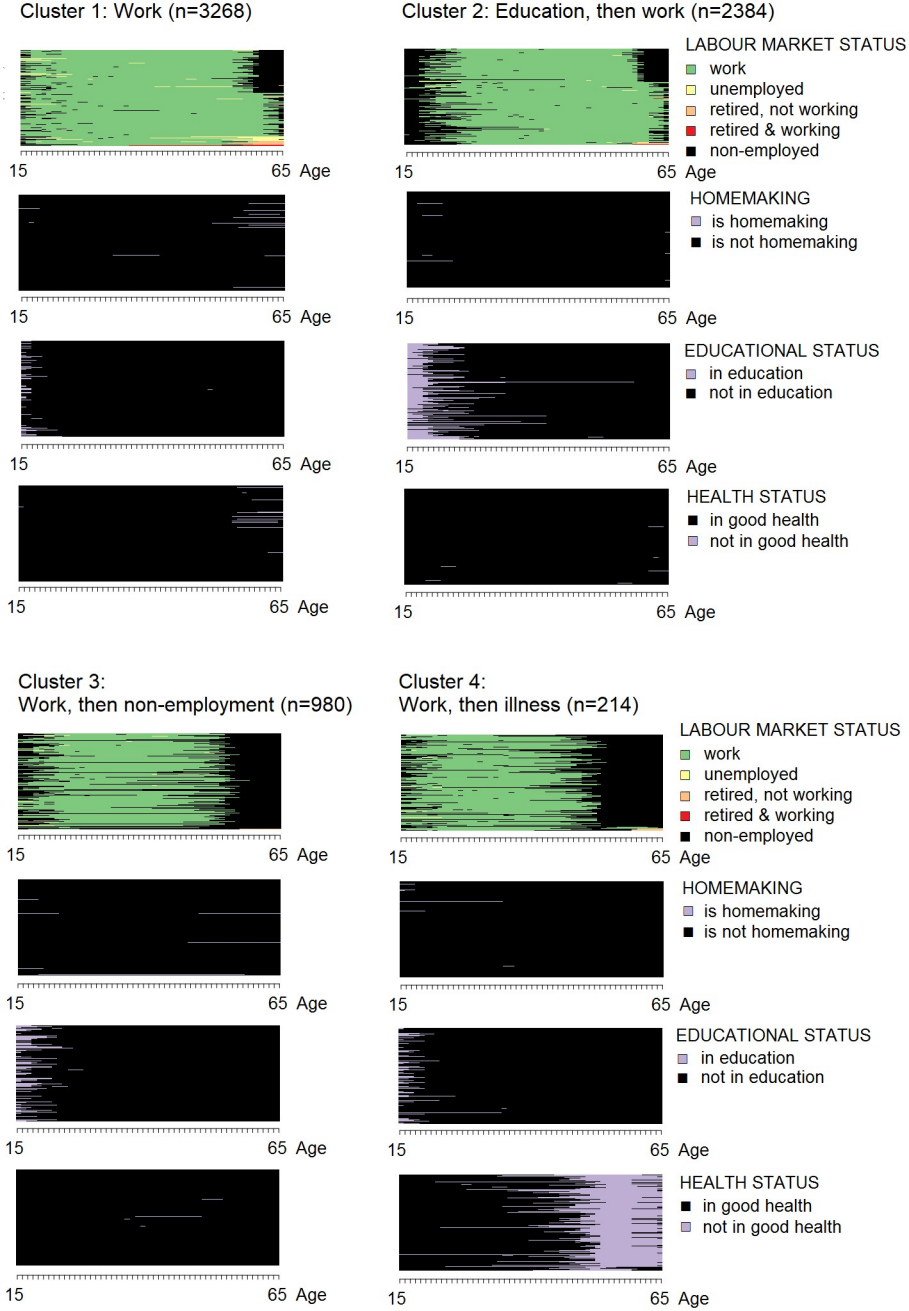
Working age life-course clusters among men.

**Fig 5:**
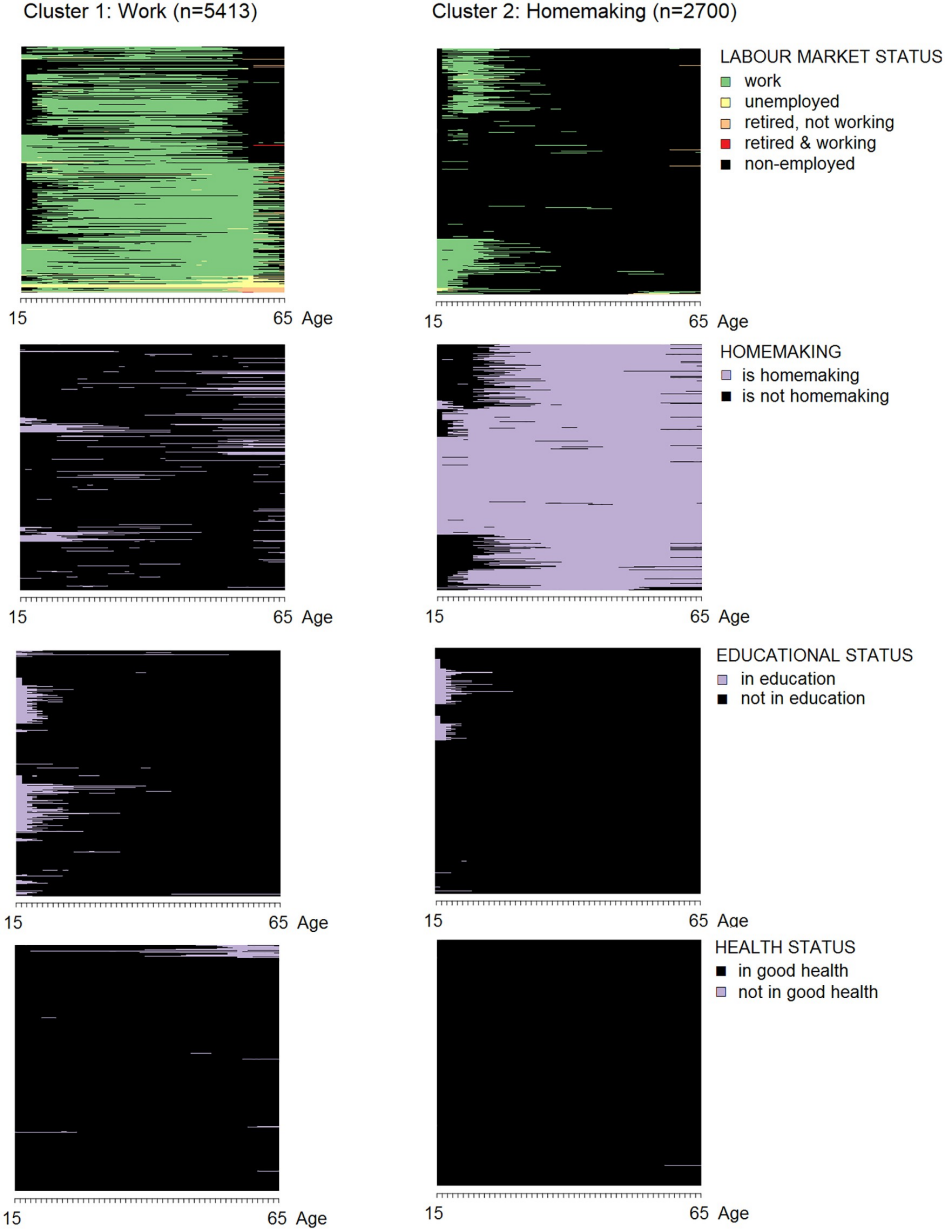
Working age life-course clusters among women.

Men’s working age life-courses cluster into four types: one that is characterized by workforce participation, one that is characterized by workforce participation following prolonged education, one of workforce participation followed by a phase of non-employment, and one of workforce participation followed by a phase of non-employment due to illness. Homemaking plays no role in any of the men’s working age life-course clusters. In contrast, women’s working age life-courses cluster into two types: one that is characterized by workforce participation and a second one that is dominated by homemaking. Some of the women in the latter cluster work for pay for a period in their teens and twenties, but the number of these women is too low to become a defining characteristic of the cluster. Educational participation and health status play no role in defining either of these clusters. The sequence plots for men are shown in Fig 4, the one for women in Fig 5.

The tripartite life-course model can capture the majority of the working age life-courses studied. In total, the working age life-courses of three out of five individuals in the sample are structured around paid work. This structure is most common in Sweden and the Czech Republic (70+ percent), and it is more common among women (67 percent) than among men (48 percent). A comparatively smaller share of working age life-courses is characterized by an initial period of education, followed by paid work (16 percent). The latter structure occurs only among men, which underlines men’s longer educational participation within the cohorts that have reached old age today. The share of men with longer educational participation is highest in Switzerland, Denmark, and Germany. Both working age life-course structures–the one characterized by work and the one characterized by education and work–are in line with the tripartite life-course model. The difference between both structures is when the transition from education to paid work occurs: One out of three men experience this transition during working age, and everybody else experiences it before working age starts. The tripartite life-course model does not assign a specific age to the transition from education to paid work, it only describes lives as a sequence of education, paid work and retirement. As such, it aligns with both working age life-course structures identified.

Overall, the tripartite life-course model captures the working age life-courses of three out of four individuals in this sample. The fit is higher among men than among women (15.8 percentage points difference), and it is higher within the younger cohort than within the older cohort (3.6 percentage points difference). It is highest in Sweden, Denmark, and the Czech Republic (88+ percent) and lowest in Greece and Spain (<63 percent; see Table 4). Most working age life-courses that are not structured around paid work are structured around homemaking (18 percent), occurring exclusively among women, more often in the older cohort and in Spain, Greece, and Ireland. Working age life-courses that contain a period of non-employment or illness after paid work occur only rarely.

**Table 4:** Working age life-course clusters by country, gender and cohort (in percent).

	In line with tripartite model			Not in line with tripartite model			
	work	education & work	total	homemaker	work & non-employed	work & ill	total
*Countries with social-democratic welfare regimes*							
Denmark	62.8	26.0	88.8	6.8	3.5	0.9	11.2
Sweden	74.1	21.2	95.3	2.4	2.0	0.3	4.7
*Countries with liberal welfare regimes*							
Ireland	54.0	9.5	63.5	29.1	6.4	1.0	36.5
Switzerland	50.3	31.5	81.8	14.9	2.7	0.6	18.2
*Countries with conservative welfare regimes*							
Austria	65.6	8.6	74.2	18.9	6.4	0.5	25.8
Belgium	51.2	16.5	67.7	22.8	8.1	1.4	32.3
France	60.4	12.7	73.1	16.1	9.4	1.4	26.9
Germany	56.1	24.5	80.6	13.6	5.0	0.8	19.4
Netherlands	56.2	12.7	68.9	21.3	5.2	4.6	31.1
*Countries with rudimentary welfare regimes*							
Greece	48.1	14.8	62.9	29.1	7.7	0.3	37.1
Italy	54.4	9.5	63.9	23.3	12.1	0.7	36.1
Spain	52.9	6.4	59.3	34.9	3.5	2.3	40.7
*Countries with post-paternalistic welfare regimes*							
Czech Republic	71.8	22.0	93.8	0.5	4.6	1.1	6.2
Poland	62.6	11.0	73.6	9.9	12.2	4.3	26.4
*By gender*							
men	47.7	34.8	82.5	0.0	14.4	3.1	17.5
women	66.7	0.0	66.7	33.3	0.0	0.0	33.3
*By birth cohort*							
until 1934	56.8	15.1	71.9	21.5	5.5	1.1	28.1
1935–44	59.0	16.5	75.5	15.4	7.4	1.7	24.5
Total	58.0	16.0	74.0	18.0	6.6	1.4	26.0
Cumulative percentage	58.0	74.0		92.0	98.6	100.0	

Table 5 shows how the welfare regimes differ in terms of working age life-courses. The tripartite model aligns with more than 90 percent of the working age life-courses in the social-democratic regime, about 80 percent in the post-paternalistic regime, about 70 percent in the conservative and liberal regimes, and about 60 percent in the rudimentary regime. The gender differences are most pronounced in the rudimentary regime, where they amount to about 40 percentage points in the cohort born until 1934 and about 30 percentage points in the younger cohort. They are least pronounced in the social-democratic regime, followed by the post-paternalistic regime, and then the liberal and conservative regimes. The fit improved and the cohort differences decreased from the older to the younger cohort in all regimes, except the post-paternalistic. In the post-paternalistic regime, the gender difference increased by 13.9 percentage points from the older to the younger cohort. The post-paternalistic and the social-democratic regimes are the only two regimes where the fit of the tripartite life-course model is better among women than among men in the younger cohort. The other regimes had a worse fit among women because a higher share of homemakers prevailed in the younger cohort. The remaining two working age life-course patterns, which contain a phase of non-employment respectively illness after work, are most common in the conservative, rudimentary, and post-paternalistic regimes. Pearson’s Chi-square tests were run for all gender, cohort, and gender-by-cohort differences within each welfare regime. These tests revealed that all differences were significant (p<0.001 for all tests).

**Table 5:** Within-country differences in working age life-course clusters, by welfare regime (in percent).

	In line with tripartite model			Not in line with tripartite model			
	work	education & work	total	homemaking	work & non-employment	work & ill	total
***Social-democratic welfare regime***							
**men, cohort 1935–1944**	39.4	51.9	91.3	0.0	7.1	1.6	100.0
**men, cohort until 1934**	47.0	48.4	95.4	0.0	3.9	0.7	100.0
**women, cohort 1935–1944**	96.0	0.0	96.0	4.0	0.0	0.0	100.0
**women, cohort until 1934**	86.5	0.0	86.5	13.5	0.0	0.0	100.0
**total**	68.8	23.4	92.2	4.5	2.7	0.6	100.0
***Liberal welfare regime***							
**men, cohort 1935–1944**	37.5	49.8	87.3	0.0	10.3	2.4	100.0
**men, cohort until 1934**	37.9	54.6	92.5	0.0	7.0	0.5	100.0
**women, cohort 1935–1944**	67.0	0.0	67.0	33.0	0.0	0.0	100.0
**women, cohort until 1934**	58.7	0.0	58.7	41.3	0.0	0.0	100.0
**total**	51.7	23.5	75.2	20.1	4.0	0.7	100.0
***Conservative welfare regime***							
**men, cohort 1935–1944**	45.6	33.9	79.5	0.0	16.0	4.5	100.0
**men, cohort until 1934**	47.0	34.8	81.8	0.0	15.0	3.2	100.0
**women, cohort 1935–1944**	70.7	0.0	70.7	29.3	0.0	0.0	100.0
**women, cohort until 1934**	59.6	0.0	59.6	40.4	0.0	0.0	100.0
**total**	56.8	15.5	72.3	18.8	7.1	1.8	100.0
***Rudimentary welfare regime***							
**men, cohort 1935–1944**	53.9	24.2	78.1	0.0	20.0	1.9	100.0
**men, cohort until 1934**	64.5	19.7	84.2	0.0	13.1	2.7	100.0
**women, cohort 1935–1944**	49.6	0.0	49.6	50.4	0.0	0.0	100.0
**women, cohort until 1934**	41.7	0.0	41.7	58.3	0.0	0.0	100.0
**total**	51.8	10.3	62.1	29.0	7.8	1.1	100.0
***Post-paternalistic welfare regime***							
**men, cohort 1935–1944**	39.4	33.0	72.4	0.0	19.8	7.8	100.0
**men, cohort until 1934**	39.0	42.9	81.9	0.0	15.6	2.5	100.0
**women, cohort 1935–1944**	92.6	0.0	92.6	7.4	0.0	0.0	100.0
**women, cohort until 1934**	88.2	0.0	88.2	11.8	0.0	0.0	100.0
**total**	67.4	16.8	84.2	5.0	8.2	2.6	100.0

## Discussion and conclusion

Life-courses are a central concept in social research. Their diversity is widely acknowledged and considered to be characteristic for societies. This article explores the structure of working age life-courses, which typically show a high degree of diversity. In doing so, it studies diversity within and across European countries.

The first research question asked which working age life-courses exist. Researchers had argued that the tripartite life-course model is overly simplistic because it reduces lives to a sequence of education, work, and retirement. Critics’ main objection is that the workforce participation may be interrupted or replaced by homemaking. Therefore, the first hypothesis was that working age life-courses are structured around paid work, around a fluctuation in and out of the labor force, or around efforts to balance work and family life. Findings showed that working age life-courses can be structured in five ways: paid work, education followed by paid work, paid work followed by non-employment, paid work followed by illness, or homemaking. Therefore, we fail to reject the first hypothesis and have to maintain it. The first two patterns identified are in line with the tripartite model, and they characterize the lives of three out of four older Europeans. This number is surprisingly high, considering that the tripartite model was derived from conceptual considerations and that it has a rather simple structure. However, the number is nevertheless too low to state that the tripartite life-course model describes life-courses in modern Western societies in general. Therefore, it seems necessary to consider the question of diverse when studying the life-courses of Europeans.

The second research question asked how working age life-courses differ across gender. Men and women are assigned different social roles, with both of them possibly working for pay and women being responsible for most homemaking. Therefore, the second hypothesis was that female working age life-courses are structured around paid work or a combination of work and homemaking, whereas male working age life-courses are structured around paid work or a fluctuation in and out of the workforce. Most women need to balance work and family life, therefore the third hypothesis is: Working age life-courses that are structured around paid work only are more common among men than among women. Findings show that men’s working age life-courses can be structured around paid work, education followed by paid work, paid work followed by non-employment, or paid work followed by illness. In contrast, women’s working age life-courses are structured around either paid work or around homemaking. In the cohorts studied, it was not yet common to combine both activities. Findings on the model fit show that the life-course patterns among women are more distinct than those among men. This finding implies that women need to make a fundamental decision on whether they want to focus on either work or homemaking. In contrast, men’s life-courses are more uniform in that they all center on paid work. Their differences stem from variation in the labour market entrance and exit. Overall, the working age life-courses of eight out of ten men and two out of three women are in line with the tripartite model. These findings suggest that there is higher diversity among men’s working age life-courses, but that this diversity is limited to a lower number of men. Women’s working age life-courses have less diversity, but a higher number of women contribute to the diversity. Consequently, the second hypothesis has to be rejected, but the third hypothesis can be maintained.

The third research question asked how working age life-courses differ across cohorts. Lives change over time, which can lead to cohort differences in working age life-courses. In the younger cohort, more women worked for pay and labor market careers became more volatile. Therefore, hypothesis 4 states that the younger cohort sees fewer women in working age life-courses that are structured around homemaking. The fifth hypothesis suggests that women’s increasing efforts to strike a work-life balance lead to new patterns of working age life-courses in the younger cohort. The sixth hypothesis states that the younger male cohort sees more fluctuation in and out of the labor market during working age. Findings highlighted that working age life-courses structured around paid work are more common within the younger cohort due to two coinciding trends: a higher share of men spend the latter part of their working age in non-employment or illness, and a lower share of women spend their working age as homemakers. The findings go beyond previously gained insight in that they show that women’s increasing workforce participation outweighs men’s difficulties in the labor market, leading to a net spreading of the tripartite life-course model. Moreover, they underline that women’s first reaction to the conundrum of having to choose between work and homemaking was to avoid homemaking–with the strategy to combine both activities only emerging in much younger cohorts [82]. Because of these findings, hypotheses four and six are maintained. However, the fifth hypothesis is rejected, because more women in the younger cohort adopt the work-centered working age life-course typical for men instead of developing new life-course patterns to better balance work and family life.

The fourth research question asked how working age life-courses differ across countries. Social context shapes how lives progress, which leads to between-country variation in life-courses. Countries with a social-democratic welfare regime have high workforce participation rates and high levels of gender equality. Consequently, the seventh hypothesis suggests that the social-democratic regime has the highest share of working age life-courses structured around paid work. Findings show that more than nine out of ten individuals in this welfare regime have a working age life-course that is spent on paid work or on education followed by paid work, which is the highest share in this sample. Consequently, the tripartite life-course model captures the work age life-courses of the vast majority of individuals in this welfare regime, and alternative working age life-course structures are of little importance. The hypothesis can be maintained.

Countries with a liberal welfare regime favor market mechanisms and accept the resulting social inequalities. The eighth hypothesis states that it has an intermediate share of individuals whose working age life-course is structured around paid work. In this regime, three out of four individuals spend their working age life-courses on paid work or on education followed by paid work. This means that an intermediate share of individuals has a working age life-course that is in line with the tripartite model. The gender and cohort differences in this regime are also of an intermediate size. Therefore, the hypothesis is maintained. This insight adds to the extant state of knowledge by highlighting the counterintuitive situation that the emphasis on market mechanisms in this regime does not lead to an above-average adoption of the tripartite life-course model, which centres on workforce participation.

In countries with a conservative regime, traditional gender roles are wide spread and women spend considerable time looking after their children. The ninth hypothesis suggests that this regime has an intermediate share of working age life-courses that are structured around paid work, and that this share is considerably lower among women. The findings parallel this hypothesis, therefore the hypothesis can be maintained. Interestingly, one country of this regime type, namely the Netherlands, has the highest share of working age life-courses containing paid work followed by a phase of illness. Poland has the second highest prevalence of this model. This finding is interesting, because the Netherlands and Poland are not among the countries with the lowest healthy life-expectancies in the sample. In fact, the healthy life-expectancies in the countries studied are so high, that poor health should not structure the working age life-courses in any of these countries [83]. It therefore seems likely that the concentration of this life-course pattern in both countries is due to the easily accessible disability schemes in both countries, which were sometimes abused as equivalents to early retirement [48, 50, 84].

Countries with a rudimentary regime adhere to the most traditional gender roles, and they place the main responsibility for childcare on women. Consequently, the tenth hypothesis states that the share of working age life-courses structured around paid work is lowest in this regime, and the share of women’s working age life-courses that are structured around homemaking is highest. Findings show that the share of individuals whose working age life-courses are structured around paid work are lowest in this regime. Although the rudimentary regime and the liberal regime tie for the lowest share of individuals who spend their entire working age on paid work, the rudimentary regime has the lowest share of individuals who receive extended education and then work for pay. At the same time, more than half the women in this regime spend their working age on homemaking, which is the highest share among all regimes. It makes this the only regime where being a homemaker is the usual activity for working age women, whereas working for pay is a deviation from the norm. This dominance of homemaking among women persists even within the younger cohort studied. The tenth hypothesis can be maintained.

Countries with a post-paternalistic regime still experience some repercussions of their communist past with its employment guarantees. The eleventh hypothesis suggests that it contains an intermediate share of working age life-courses that are structured around paid work, and this share is considerably higher in the older cohort. Findings are in line with this hypothesis, and the hypothesis needs to be maintained. However, findings also reveal additional details on within-country differences. They show that the share of working age life-courses that are structured around paid work is higher among the older male cohort, but not among the older female cohort. Therewith, the gender-specific cohort differences follow the general trend found in all regimes, whereas the cohort-difference for the entire population follows a regime-specific trend. The coincidence of the regime-specific and the general trend in cohort differences leads to a unique situation: the post-paternalistic regime is the only regime where the share of working age life-courses that are structured around paid work is higher among women than among men–with the gender-difference even growing across cohorts.

**Fig 6:**
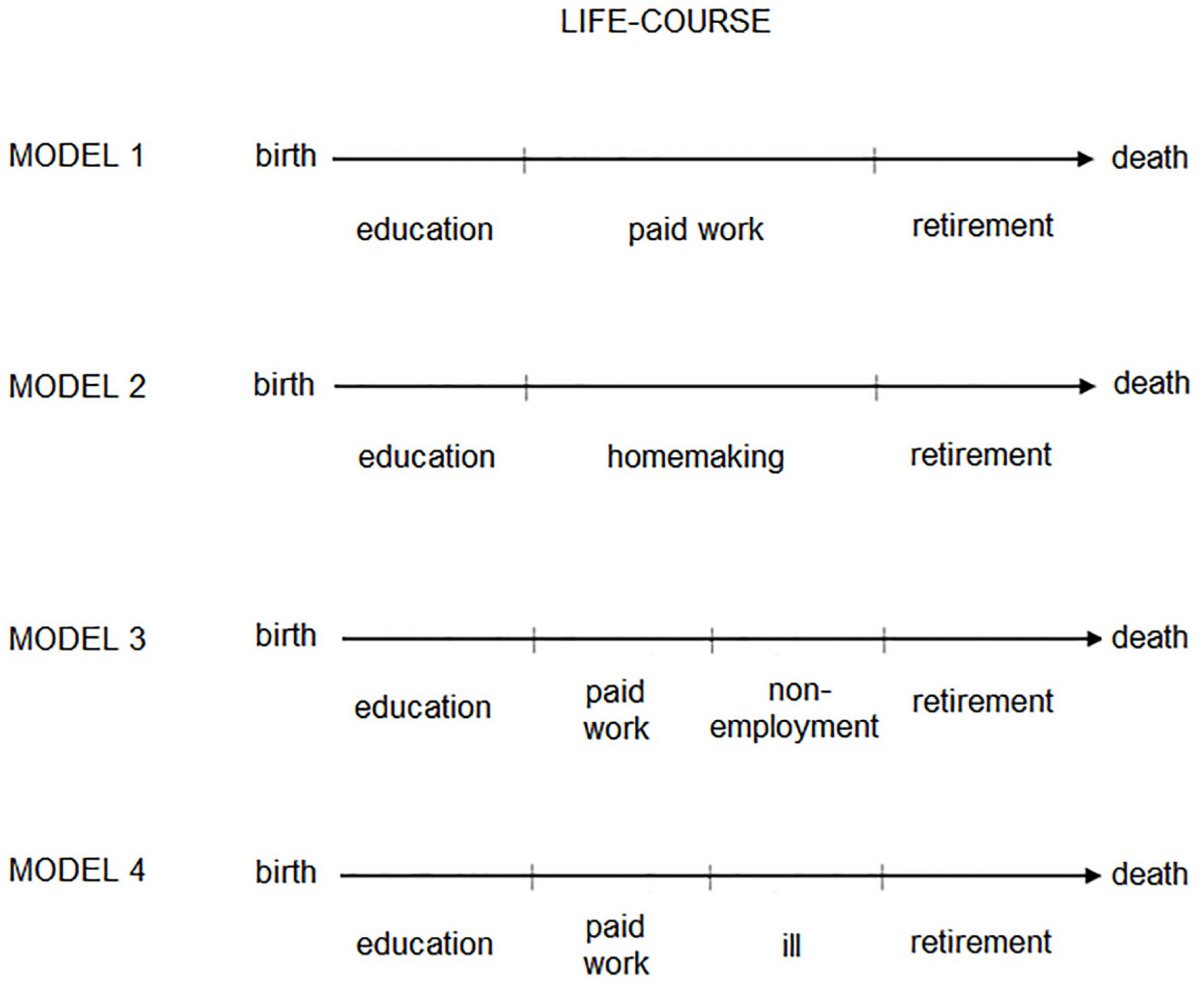
The life-course models suggested by the data.

Findings have theoretical and practical implications. Theoretical implications arise because findings enhance our understanding of life-course structures and the diversity among them. The vast majority of working age life-courses display a structure that is in line with the tripartite life-course model, which describes lives as a sequence of education, work, and retirement. Therefore, the fit of this model is much better than what current academic discussions suggest. Consequently, researchers can use the tripartite life-course model in their studies with more confidence. Considering that the fit of the model is better in the younger cohort, it may become even more useful in the years and decades to come. Nevertheless, this study also reveals the existence of diversity in working age life-courses, within and between countries. Fig 6 shows how this diversity can be captured in a set of four life-course models: the tripartite model; a model that describes middle age as a time of homemaking; a model that describes middle age as a time of work followed by a period of non-employment; and a model that describes middle age as a time of work followed by a period of illness.

Researchers can use this set of life-course models to describe differences in how lives across Europe progress. The between-country differences identified align with the welfare regime–specific life-courses outlined by Mayer [20, 26]. Therefore, the findings of this article can be used to refine Mayer’s [20, 26] descriptions and to add a post-paternalistic regime to his typology, which did not consider the situation in Eastern Europe.

For a parsimonious description of life-courses, the use of two models suffices. A combination of the tripartite model and the model assigning middle age to homemaking can capture the lives of more than nine out of ten older Europeans. Interestingly, none of the models includes simultaneously occurring activities. Some researchers, such as Riley and Riley [76], suggest that such simultaneity may become increasingly common within Western life-courses. This study did not find any evidence for this suggestion. However, the increase may by typical for cohorts younger than the ones studied in this article. Recent research discussed especially women’s increasing efforts to strike a balance between work and homemaking [41, 62, 63, 82]. Consequently, the new life-course patterns may be characteristics for those cohorts of women that are currently having children. Among them, new and more complex life-course patterns may emerge, replacing especially the old pattern of homemaking only. The current study can serve as a point of comparison when observing newly emerging life-course patterns. Moreover, it provides models for estimating what newly emerging life-course patterns could be. Most notably, recent studies pointed to increasing unemployment due to the 2008 recession and the on-going social change in Eastern Europe [53, 65, 66]. In the wake of the 2008 crisis, unemployment rates increased especially among youths, who could not establish themselves in the labor market, and among older individuals, who sometimes responded with early retirement. This observation is in line with the findings of this study, which documented that the variety in work-centered life-courses lie mainly in the workforce entrance and exit. The increasing unemployment rates may establish new life-course patterns that contain phases of unemployment in between leaving education and starting to work, and in between work and retirement. Such life-course patterns are most likely to emerge in Southern and Eastern Europe over the coming years. The reasons are that especially Southern European countries suffered during the 2008 crisis [53], and that Eastern European countries are still transforming as they transition into market economies.

Practical implications arise because dependency ratios are calculated using the logic of a tripartite life-course model. Dependency ratios are common indicators for the age structure in a population and for the reform pressure on pension schemes [21, 22]. The ratios are calculated using the structure of the tripartite life-course model, which means that they can only be as accurate as the tripartite life-course model itself. This study highlights the diversity of life-courses across Europe, and underlines differences between cohorts and countries. As a result, time series and country comparisons with dependency ratios need to be interpreted with caution, to make sure that one is not comparing the proverbial apples and oranges. As such, policy recommendations on pension reforms in Southern and Central Europe and the Anglo-Saxon countries that were formulated because of historical change in dependency ratios should be treated with caution. In older cohorts, the dependency ratio gives inaccurate information on the ratio between workers and pensioners, because it is built on wrong assumptions about many of the women’s life-courses. However, the fit of the tripartite life-course model in these countries improved in younger cohorts because of women streaming into the labor market. As a result, the pressure on pension schemes exerted by increasing dependency ratios in these countries may be at least partly offset by women’s increasing workforce participation.

Despite its merits, this study also has some limitations. First, it focuses on activities between the ages of 15 and 65 only. These ages were chosen because they are considered the working age, where people experience most changes and fluctuations in activities. However, deviations in life-course structures can also occur before the age of 15 and after the age of 65 [5, 85]. Further studies will need to test changes in these additional age groups and further modify the life-course models, if needed. Second, this study analyzes data on cohorts that had already reached the age of 65 years. This approach allows for studying the entire time span from 15 to 65 years, which is a novel approach and an important contribution to the scientific body of knowledge. The downside is that this approach does not capture the life-courses of cohorts that are younger than 65 years. Additional studies are needed to test for life-course changes across cohorts. Finally, this study analyzed data from only those people who survived until age 65 and participated in the survey. Individuals who died before this age or who were too frail to participate in the survey were not included. As a result, this study does not capture life-courses characterized by an early death, and it may underestimate the role of illness in early old age. Additional studies focusing on these phenomena are needed to determine whether further working age life-course patterns exist.

Overall, this study contributes to our understanding of life-courses and social inequalities. It demonstrates the prevalence of the tripartite life-course model across Europe. This model fits working age life-courses much better than current scientific discussions suggest, and its fit may still improve over time. This study also highlights diversity among working age life-courses, showing that within-country differences strongly align with gender and cohort: Working age life-courses spent on homemaking are especially prevalent among women in the older cohort, whereas working age life-courses spent on paid work followed by illness or non-employment are especially prevalent among men. Finally, this study outlines between-country differences in working age life-courses, pointing out a North-South decline in the fit of the tripartite life-course model. Researchers interested in country differences can use the life-course models developed to better describe welfare regime-specific life-course patterns. This perspective makes working age life-courses a property of societies, which can be used to map social inequalities and capture social change over time.
